# Local impedance measurements during contact force‐guided cavotricuspid isthmus ablation for predicting an effective radiofrequency ablation

**DOI:** 10.1002/joa3.12680

**Published:** 2022-02-04

**Authors:** Takehito Sasaki, Kohki Nakamura, Kentaro Minami, Yutaka Take, Yosuke Nakatani, Yuko Miki, Koji Goto, Kenichi Kaseno, Eiji Yamashita, Keiko Koyama, Shigeto Naito

**Affiliations:** ^1^ Division of Cardiology Gunma Prefectural Cardiovascular Center Maebashi City Japan; ^2^ Division of Radiology Gunma Prefectural Cardiovascular Center Maebashi City Japan

**Keywords:** atrial flutter, cavotricuspid isthmus, contact force, local impedance, radiofrequency catheter ablation

## Abstract

**Background:**

An ablation catheter capable of contact force (CF) and local impedance (LI) monitoring (IntellaNav StablePoint, Boston Scientific) has been recently launched. We evaluated the relationship between the CF and LI values during radiofrequency catheter ablation (RFCA) along the cavotricuspid isthmus (CTI).

**Methods:**

Fifty consecutive subjects who underwent a CTI‐RFCA using IntellaNav StablePoint catheters were retrospectively studied. The initial CF and LI at the start of the RF applications and mean CF and minimum LI during the RF applications were measured. The absolute and percentage LI drops were calculated as the difference between the initial and minimum LIs and 100 × absolute LI drop/initial LI, respectively.

**Results:**

We analyzed 602 first‐pass RF applications. A weak correlation was observed between the initial CF and LI (*r* = 0.13) and between the mean CF and LI drops (*r* = 0.22). The initial LI and absolute and percentage LI drops were greater at effective ablation sites than ineffective ablation sites (median, 151 vs. 138 Ω, 22 vs. 14 Ω, and 14.4% vs. 9.9%; *p* < .001), but the initial and mean CF did not differ. At optimal cutoffs of 21 Ω and 10.8% for the absolute and percentage LI drops according to the receiver‐operating characteristic analysis, the sensitivity, and specificity for predicting an effective ablation were 57.4% and 88.9% and 80.0%, and 61.1%, respectively.

**Conclusions:**

The effective sites during the CF‐guided CTI‐RFCA had greater initial LI and LI drops than the ineffective sites. Absolute and percentage LI drops of 21 Ω and 10.8% may be appropriate targets for an effective ablation.

## Graphical abstract

During radiofrequency catheter ablation (RFCA) along the cavotricuspid isthmus (CTI) using an ablation catheter capable of simultaneous contact force (CF) and local impedance (LI) monitoring, the effective ablation sites exhibited higher initial local impedance (LI) values and greater LI drops than the ineffective ablation sites. The receiver operating characteristic analysis suggested that an absolute LI drop of 21 Ω and percentage LI drop of 10.8% may be suitable targets for an effective ablation during a CTI‐RFCA.

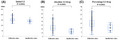

## 1 INTRODUCTION

Radiofrequency catheter ablation (RFCA) along the cavotricuspid isthmus (CTI) is a commonly utilized procedure for treating CTI‐dependent atrial flutters.^1^, ^2^ The goal of a CTI‐RFCA is to complete bidirectional conduction block along the CTI.^3^ Several indices for contributing to successful completion of the CTI bidirectional conduction block have been reported: the contact force (CF),^4^ force‐time integral (FTI),^5^ ablation index (AI) incorporating the CF, RF application duration, and power in a weighted formula,^6^, ^7^ and electroanatomic coupling index.^8^

Local impedance (LI) monitoring during RFCA has been reported to be useful for predicting an effective ablation,^9^, ^10^ and greater LI drops are associated with an effective lesion formation.^11^, ^12^, ^13^ In those previous studies with LI monitoring during RFCA, the LI values were measured using a 4‐electrode method with micro‐electrodes on the 4.5‐mm tip of the ablation catheter (IntellaNav MiFi OI, Boston Scientific). On the other hand, a newer ablation catheter capable of LI monitoring (IntellaNav StablePoint; Boston Scientific), which has been recently introduced to clinical practice, uses a 3‐electrode method without micro‐electrodes to measure the LI values and has a 4‐mm catheter tip.^14^ In addition, the newer LI‐monitoring ablation catheter is also capable of CF monitoring. As a result of the difference in the LI measurement methods, size of the catheter tip, and presence or absence of CF monitoring, the LI values, and drops during a CTI‐RFCA are likely to differ between those two types of LI‐monitoring ablation catheters. So far there have been no published clinical studies on CTI‐RFCA using an ablation catheter capable of simultaneous CF and LI monitoring. Thus, we sought to investigate the relationship between the CF and LI values during a CTI‐RFCA using the newer LI‐monitoring ablation catheter and compared those values between the effective and ineffective ablation sites.

## 2 METHODS

### 2.1 Study population

Fifty consecutive subjects who underwent a first CTI‐RFCA and RFCA of atrial fibrillation (AF) at Gunma Prefectural Cardiovascular Center from December 2020 to July 2021 were included in this retrospective observational study. All RFCA procedures were performed using the IntellaNav StablePoint ablation catheter with the Rhythmia mapping system (Boston Scientific). Eleven subjects (22.0%) had CTI‐dependent atrial flutters before the RFCA procedure.

Oral anticoagulation was started more than 1 month prior to the procedure and was continued throughout the periprocedural period. All class I antiarrhythmic drugs were stopped pre‐procedurally for ≥5 half‐lives, while bepridil was continued during the periprocedural period. All subjects gave written informed consent, and the study protocol was approved by the local ethics committee and adhered to the Declaration of Helsinki.

### 2.2 RFCA

We previously published the RFCA procedures and periprocedural anticoagulation protocol.^13^, ^15^ During the procedure, propofol or dexmedetomidine, and pentazocine were administered intravenously to maintain deep conscious sedation. The activated clotting time was maintained between 300 and 400 s by an intravenous continuous and bolus infusion of heparin throughout the procedure. A multielectrode catheter was positioned in the coronary sinus (CS) via the right femoral vein and served as the positional reference for mapping using the Rhythmia system. A steerable sheath (Agilis NxT; Abbott) was advanced into the right atrium (RA), and the ablation catheter was introduced through the sheath. The CTI linear ablation was performed with a point‐by‐point ablation from the tricuspid annulus to the inferior vena cava during CTI‐dependent atrial flutters or pacing from the proximal CS. Continuous RF lesions were created along the CTI with an inter‐lesion distance of 4–5 mm between two neighboring lesions. Each application of RF energy was delivered with a target CF of 5–20 g, power output of 25–40 W, RF duration of 20–40 s, and maximum temperature of 40°C at an irrigation flow rate of heparinized saline of 8 or 15 ml/min. The RF energy deliveries were started when the ablation catheter tip was positioned on the target ablation site and the CF values reached ≥5 g and were stopped when the LI values reached the plateau of the impedance curve, decreased to >50 Ω from that when starting the RF applications, or increased steadily during ongoing ablation.^14^ If the ablation catheter tip was displaced during the ongoing ablation, the RF energy deliveries were immediately stopped and applied again at the same site after repositioning the ablation catheter tip. When the LI values did not sufficiently drop during the ongoing ablation, the RF power output was increased up to 40 W or the catheter orientation relative to the ablation site (parallel, oblique, or perpendicular) was changed at the operators' discretion. When the LI values rapidly dropped, the RF power output was decreased.

First‐pass conduction block was defined as the completion of bidirectional conduction block by the first linear ablation from the tricuspid valve annulus to the inferior vena cava without any additional RF applications. When the first‐pass block was not achieved or the recovery of conduction occurred during the waiting period, the conduction gaps were mapped using the ablation catheter or a 64‐electrode mini‐basket catheter (IntellaMap Orion, Boston Scientific), and additional RF ablation was applied to eliminate the gaps. During the additional RF applications at sites with gaps, the catheter orientation relative to those sites was changed from that during the initial RF applications, so that the LI values would further drop. The completion of bidirectional conduction block along the CTI was verified by standard electrophysiological methods, including differential pacing maneuvers and recording widely spread double potentials along the CTI ablation line.^16^, ^17^

### 2.3 Procedural parameters

**FIGURE 1:**
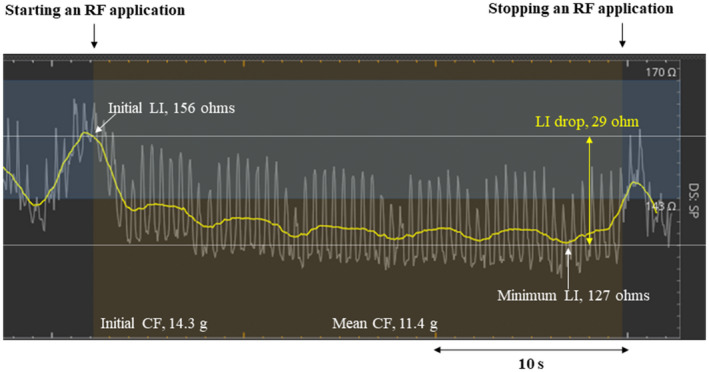
Real‐time LI curves during the RF ablation. The yellow and white curves indicate the mean LI values for the last 3 s and raw data of the LI values, respectively. The initial and minimum LI values during the RF application were 156, and 127 ohms, respectively, and the absolute and percentage LI drops were 29 ohms and 18.6%, respectively. CF, contact force; LI, local impedance

Effective ablation sites were defined as those sites without conduction gaps during the first‐pass CTI‐RFCA, while ineffective ablation sites were defined as those with conduction gaps. The following procedural parameters during the first‐pass CTI‐RFCA were evaluated: the CF, RF power output, RF application duration, RF energy delivery, FTI, and LI. The initial and mean CF were defined as the CF values measured at the start of the RF applications and the average value of the CF during the RF applications, respectively. The FTI was calculated by multiplying the mean CF by the RF application duration. The LI was measured using a 3‐electrode method, by driving a non‐stimulatory alternating current (5.0 μA at 14.5 kHz) between the tip electrode and proximal ring to create a local potential field.^14^ The initial and minimum LIs were defined as the LI value measured at the start of the RF applications and minimum value during the RF applications, respectively (Figure 1). Further, the absolute and percentage LI drops were obtained as the difference between the initial and minimum LIs and 100 × absolute LI drop/initial LI, respectively.

### 2.4 Statistical analysis

Normally distributed continuous variables were expressed as the mean ± standard deviation (SD), non‐normally distributed continuous variables were as the median and interquartile range (IQR, 25th–75th percentile), and categorical variables were as the number and percentage of subjects. A Mann–Whitney's *U*‐test was used for a comparative analysis between the effective and ineffective ablation sites during the first‐pass CTI‐RFCA. The relationship between the CF values and LI drops was evaluated using Pearson's correlation coefficient. To compare the strength of the association of the parameters for predicting an effective RF ablation, the area under the receiver operating characteristics (ROC) curve for each parameter was estimated. The statistical significance for all tests was accepted at a *p*‐value of *<*0.05. The statistical analyses were performed using JMP 11.2 software (SAS Institute Inc.,).

## 3 RESULTS

Tables 1 and 2 present the baseline and procedure‐related characteristics of the study subjects. The mean age was 67 ± 11 years, and 35 subjects (70.0%) were men. Thirty‐two subjects (64.0%) had first‐pass CTI conduction block, while the remaining 18 (36.0%) had some residual conduction gaps after the first‐pass CTI ablation. No recovery of conduction along the CTI occurred during the waiting period in the study subjects. Finally, CTI bidirectional conduction block was completed in all subjects. No procedure‐related complications occurred in this series, including an audible steam pop, cardiac tamponade, or stroke. During a median follow‐up period of 169 days (IQR, 109–231 days) after the RFCA procedure, none of the subjects had any recurrences of CTI‐dependent atrial flutters.

**TABLE 1:** Baseline characteristics of the study subjects

Number of subjects	50
Age (years)	67 ± 11
Male gender, *n* (%)	35 (70)
Body mass index (kg/m^2^)	24.0 (21.1–26.5)
Hypertension, *n* (%)	30 (60)
Diabetes mellitus, *n* (%)	14 (28)
Heart failure, *n* (%)	13 (26)
Stroke/transient ischemic attack, *n* (%)	3 (6)
CHADS_2_ score	2 (1–2)
Transthoracic echocardiographic findings	
Left atrial diameter (mm)	42 (37–46)
Left ventricular ejection fraction (%)	60 (45–65)

Note: CHADS_2_, congestive heart failure, hypertension, age ≥ 75 years, diabetes mellitus (1 point for the presence of each), and a stroke and transient ischemic attack (2 points).

**TABLE 2:** Procedure‐related characteristics during the CTI‐RFCA

Procedure time (min)	10.5 (7.0–13.3)
Fluoroscopy duration (min)	3.4 (2.1–5.4)
Number of RF applications	11 (9–14)
Total RF duration (s)	408 (340–485)

Abbreviations: RF, radiofrequency.

### 3.1 CF and LI analyses during the first‐pass CTI‐RFCA

A total of 602 RF applications during the first‐pass CTI‐RFCA, including 584 effective RF applications and 18 ineffective RF applications, were analyzed (Table 3). The LI values reached the plateau of the impedance curve in 579 (99.1%) of the effective RF applications and 18 (100%) of the ineffective RF applications, respectively. On the other hand, the LI values continued declining during the ongoing ablation in five (0.9%) of the effective RF applications and none of the ineffective RF applications, respectively, and those RF applications were stopped when the LI values decreased to >50 Ω. No RF applications exhibited an LI rise during the ongoing ablation.

**TABLE 3:** Comparison of the procedure‐related parameters between the effective and ineffective ablation sites during the first‐pass CTI‐RFCA

	Effective sites (*n* = 584)	Ineffective sites (*n* = 18)	*p* value
Maximum RF power output (W)	35 (30–36)	35 (30–36)	0.39
RF application duration (s)	30 (28–34)	30 (29–31)	0.93
RF energy delivery (J)	910 (762–1070)	880 (728–1006)	0.55
Initial CF (g)	8.3 (5.6–11.8)	9.5 (5.7–13.7)	0.48
Mean CF (g)	9.6 (7.3–13.0)	10.6 (8.4–14.0)	0.27
FTI (gs)	288.0 (216.6–374.4)	342.2 (241.6–453.8)	0.17
Initial LI (Ω)	151 (142–164)	138 (135–147)	0.0003
Minimum LI (Ω)	129 (122–137)	125 (116–134)	0.10
Absolute LI drop (Ω)	22 (17–29)	14 (10–19)	<0.0001
Percentage LI drop (%)	14.4 (11.5–18.2)	9.9 (6.9–13.4)	<0.0001

Abbreviations: CF, contact force; FTI, force‐time Integral; LI, local impedance; RF, radiofrequency.

**FIGURE 2:**
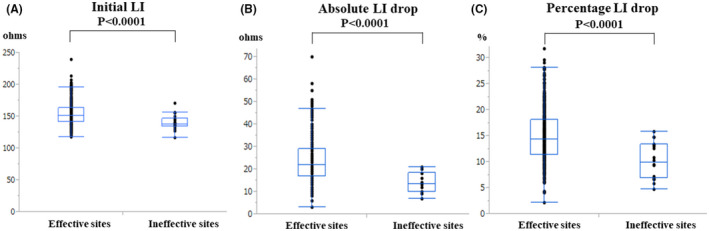
(A) Initial LI values, (B) absolute LI drops, and (C) percentage LI drops at the effective and ineffective ablation sites during the CTI‐RFCA. The initial LI values and absolute and percentage LI drops are shown by the black dots. In the box‐and‐whisker plots, the horizontal lines with boxes and bars represent the median with interquartile range and maximum and minimum values (excluding outliers), respectively. LI, local impedance

The initial and mean CF, maximum RF power output, RF application duration, RF energy delivery, and FTI did not significantly differ between the effective and ineffective ablation sites. A weak correlation was observed between the initial CF and LI values (*r* = 0.13, *p* = 0.0015). The effective ablation sites exhibited significantly higher initial LI values than the ineffective ablation sites (median, 151 Ω [IQR, 142–164 Ω] vs. median, 138 Ω [IQR, 135–147 Ω], *P* = 0.0003) (Figure 2A).

**FIGURE 3:**
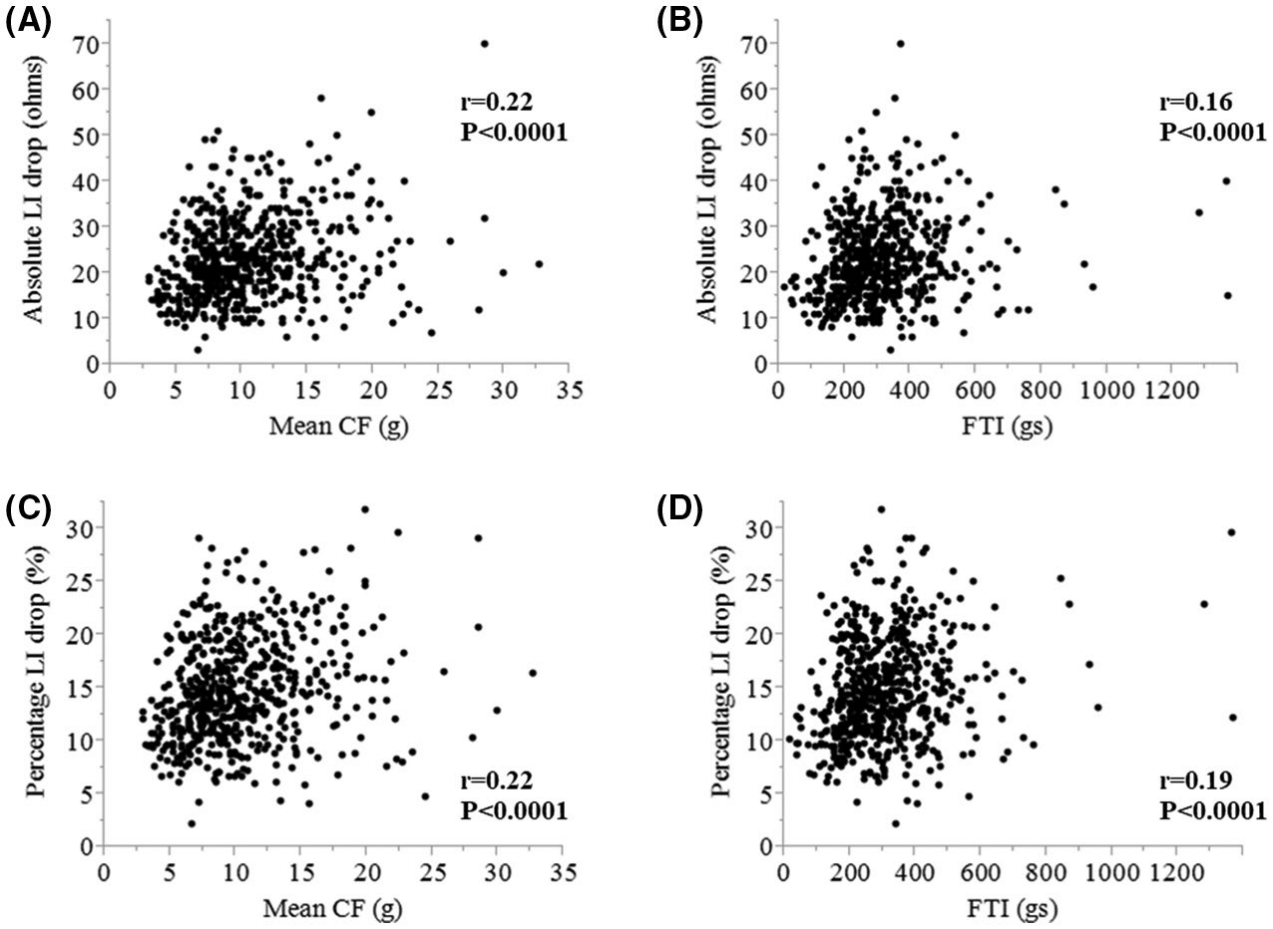
Scatter plots between the (A) mean CF and absolute LI drop, (B) FTI and absolute LI drops, (C) mean CF and percentage LI drop, and (D) FTI and percentage LI drop for all RF applications. The *r* value in each scatter diagram indicates the correlation coefficient. CF, contact force; LI, local impedance; FTI, force‐time integral

The absolute and percentage LI drops were significantly greater at the effective ablation sites than ineffective ablation sites (median, 22 Ω [IQR, 17–29 Ω] vs. median, 14 Ω [IQR, 10–19 Ω], *p* < 0.0001; median, 14.4% [IQR, 11.5%–18.2%] vs. median, 9.9% [IQR, 6.9%–13.4%], *p* < 0.0001) (Figure 2B,C). A weak correlation was observed between the mean CF value and absolute LI drop (*r* = 0.22, *p* < 0.0001), mean CF value and percentage LI drop (*r* = 0.22, *p* < 0.0001), FTI and absolute LI drop (*r* = 0.16, *p* < 0.0001), and FTI and percentage LI drop (*r* = 0.19, *p* < 0.0001) (Figure 3).

**FIGURE 4:**
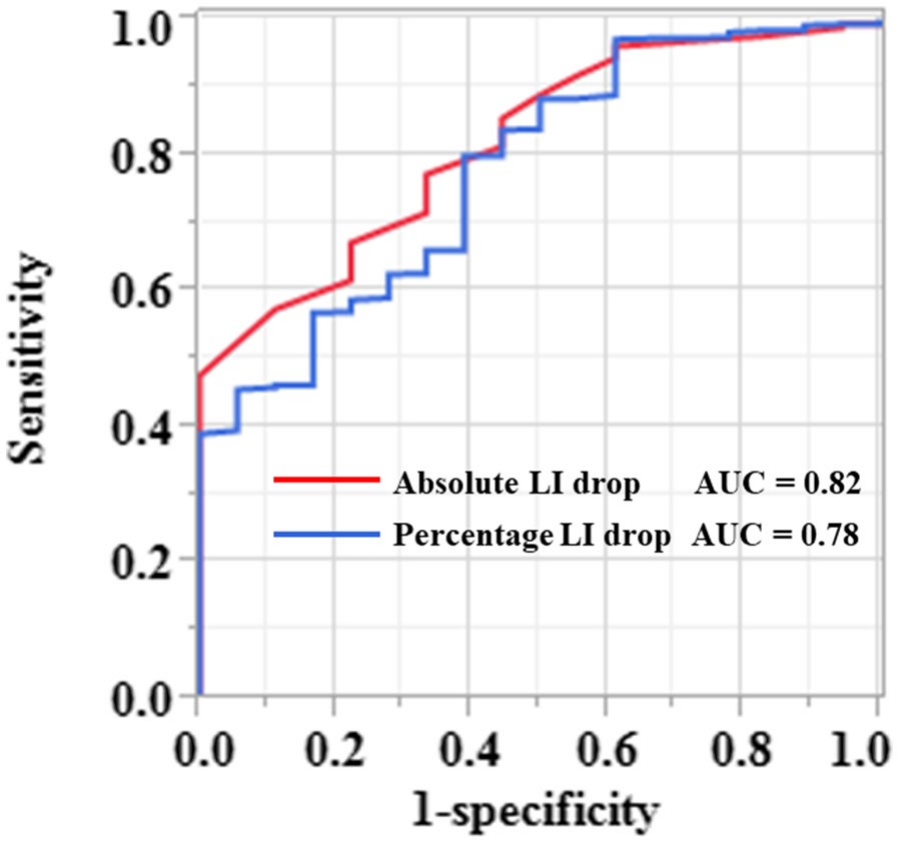
Receiver operating characteristic curves for the absolute (red line) and percentage (blue line) LI drops as predictors of an effective RF ablation during the CTI‐RFCA. AUC, area under the curve; LI, local impedance

An ROC analysis demonstrated that the area under the curve (AUC) was 0.82 (95% confidence interval [CI], 0.73–0.89) for the absolute LI drop and 0.78 (95% CI, 0.66–0.86) for the percentage LI drop, respectively, and the absolute LI drop had a significantly larger AUC than the percentage LI drop (*p* = 0.01) (Figure 4). According to the results of the ROC analysis, the sensitivity and specificity for predicting the effectiveness of ablation were 57.4% and 88.9% at an optimal cutoff of 21 Ω for the absolute LI drop and 80.0% and 61.1% at an optimal cutoff of 10.8% for the percentage LI drop, respectively. The LI values reached the plateau of the impedance curve without an absolute LI drop of ≥21 Ω at 249 (42.6%) out of 584 effective ablation sites and 16 (88.9%) out of 18 ineffective ablation sites, respectively (*p* = 0.0001), while the LI values reached the plateau of the impedance curve without a percentage LI drop of ≥10.8% at 117 effective ablation sites (20.0%) and 11 ineffective ablation sites (61.1%), respectively (*p* = 0.0002).

Comparison of the procedure‐related parameters between the initial and additional RF applications at the ineffective ablation sites during the first‐pass CTI‐RFCA.

Table 4 presents a comparison of the procedure‐related parameters between the initial and additional RF applications at the 18 ineffective ablation sites during the first‐pass CTI‐RFCA. The initial and mean CF, maximum RF power output, RF application duration, RF energy delivery, FTI, and initial LI values did not significantly differ between the initial and additional RF applications. The absolute and percentage LI drops were significantly greater during the additional RF applications than initial RF applications (median, 16 Ω [IQR, 13–21 Ω] vs. median, 14 Ω [IQR, 10–19 Ω], *p* = 0.009; median, 12.2% [IQR, 9.5%–14.8%] vs. median, 9.9% [IQR, 6.9%–13.4%], *p* = 0.01).

**TABLE 4:** Comparison of the procedure‐related parameters between the initial and additional RF applications at the 18 ineffective ablation sites during the first‐pass CTI‐RFCA

	Initial RF applications	Additional RF applications	*p* value
Maximum RF power output (W)	35 (30–36)	35 (30–35)	0.72
RF application duration (s)	30 (29–31)	30 (29–33)	0.29
RF energy delivery (J)	880 (728–1006)	827 (664–1071)	0.70
Initial CF (g)	9.5 (5.7–13.7)	10.0 (5.8–14.9)	0.38
Mean CF (g)	10.6 (8.4–14.0)	10.7 (7.7–15.0)	0.96
FTI (gs)	342.2 (241.6–453.8)	349.5 (228.2–486.1)	0.86
Initial LI (Ω)	138 (135–147)	138 (130–146)	0.48
Minimum LI (Ω)	125 (116–134)	122 (113–130)	0.05
Absolute LI drop (Ω)	14 (10–19)	16 (13–21)	0.009
Percentage LI drop (%)	9.9 (6.9–13.4)	12.2 (9.5–14.8)	0.01

Abbreviations: CF, contact force; FTI, force‐time integral; LI, local impedance; RF, radiofrequency.

## 4 DISCUSSION

### 4.1 Major findings

This retrospective observational study investigated the relationship between the CF and LI values during a CTI‐RFCA using an ablation catheter capable of simultaneous CF and LI monitoring with the Rhythmia mapping system. We demonstrated that the LI at the start of the RF applications was significantly higher at the effective ablation sites than ineffective ablation sites, while the CF at the start of the RF applications did not significantly differ between those sites. Thus, a weak correlation was observed between the initial CF and LI values. Further, the LI drops were significantly greater at the effective ablation sites than ineffective ablation sites, while the mean CF and FTI during the RF applications did not significantly differ between those sites. A weak correlation was observed between the mean CF and LI drops and the FTI and LI drops. The ROC analysis suggested that an absolute LI drop of 21 Ω and percentage LI drop of 10.8% may be appropriate targets for an effective RF ablation during a CTI‐RFCA.

### 4.2 CF and LI monitoring during RFCA

The RF catheter tip‐tissue contact significantly impacts the size and transmurality of RF lesions and the clinical outcomes of RFCA procedures.^18^, ^19^ Real‐time CF measurements during RF applications contribute to more a stable and efficient catheter‐tissue contact.^4^ Thus, the use of CF‐sensing ablation catheters has been widespread in RFCA procedures and novel markers of the ablation lesion quality such as the AI and lesion size index have been developed.^20^, ^21^ On the other hand, the LI also provides information on the catheter‐tissue coupling, issue characteristics, and tip electrode surface area covered by the myocardium. Previous studies reported that LI drops could predict the lesion formation during RF ablation and were significantly greater at effective ablation sites than ineffective ablation sites.^9^, ^10^, ^11^, ^12^, ^13^ However, the CF‐related information was not available in those studies because the LI‐monitoring ablation catheter (IntelaNav MiFi OI, Boston Scientific) used was not capable of CF monitoring. In addition, the newer LI‐monitoring ablation catheter (IntellaNav StablePoint, Boston Scientific) has a different LI measurement method than the former LI‐monitoring ablation catheter used in those studies: 3‐electrode method without micro‐electrodes versus 4‐electrode method with micro‐electrodes.^9^, ^14^ Further, those ablation catheters have different sizes of the catheter tips: 4.5 mm for the former ablation catheter versus 4 mm for the newer ablation catheter. Therefore, the LI data obtained from those studies with the former ablation catheter cannot be applied to the newer ablation catheter capable of simultaneous CF and LI monitoring. Actually, the initial LI tended to be higher and LI drops tended to be greater in the current study than our previous study with a CTI‐RFCA using the former LI‐monitoring ablation catheter.^13^

During the CTI‐RFCA in the current study, the effective ablation sites exhibited a significantly higher initial LI and greater LI drop than the ineffective ablation sites. In an experimental study using the IntellaNav StablePoint catheter by Garrott et al., the LI drop strongly correlated with the lesion depth.^14^ Szegedi et al. reported that during a pulmonary vein (PV) isolation using the IntellaNav StablePoint catheter, the successful RF applications had a higher initial LI and larger LI drop than unsuccessful RF applications,^22^ which was consistent with the results of the current study. On the other hand, the initial LI values were comparable between the effective and ineffective ablation sites in our previous study with a CTI‐RFCA using the IntelaNav MiFi OI catheter, although the LI drops were significantly greater at effective ablation sites than ineffective ablation sites.^13^ A possible explanation for the difference in the results may be the presence or absence of CF monitoring. As a result of the presence of CF monitoring in the current study, the catheter‐tissue contact may have been more stable and constant.

### 4.3 Relationship between the CF and LI during the CTI‐RFCA

The LI depends on not only the catheter‐tissue contact but also several factors: the tissue characteristics, catheter orientation relative to the ablation site (parallel, oblique, or perpendicular), and tip electrode surface area covered by the myocardium.^9^, ^11^, ^14^, ^23^ This may be why the initial CF hardly correlated with the initial LI in the current study. Further, the mean CF and FTI were comparable between effective and ineffective ablations sites, and the LI drops hardly correlated with the mean CF and FTI. That may be because those factors that can affect the LI values mentioned above have an impact on LI drops during RF ablation. Since the CTI often has a complex anatomy, such as a pouch, not only the magnitude of the CF but also the catheter tip‐tissue coupling is important to achieve an effective RF ablation. Therefore, simultaneous CF and LI monitoring appeared to be useful to improve the catheter tip‐tissue coupling and consequently the RF lesion formation by directly providing biophysical feedback.

### 4.4 LI drops for predicting an effective ablation during the CTI‐RFCA

The transmurality and continuity of the RF lesions are essential for creating bidirectional conduction block along the CTI.^6^, ^8^ The current study suggested that the optimal absolute and percentage LI drops for an effective RF ablation during the CTI‐RFCA were ≥21 Ω and ≥10.8%, respectively. Szegedi et al demonstrated that an LI drop of >22 Ω along the anterior PV isolation line and >18 Ω on the posterior PV isolation line might be useful cut‐offs for determining an optimal lesion formation.^22^ Those target LI values during the PV isolation were similar to those during the CTI‐RFCA in the current study.

Excessive ablation increases the risk of audible steam pops and char formation.^18^ Thus, it is critical to identify not only the minimum target of the LI drops for creating transmural lesions but also maximum target for preventing an excessive ablation during a CTI‐RFCA. Nevertheless, no RF applications had any audible steam pops or charring on the ablation catheter tip and the maximum target of the LI drops remained unclear in the current study. Garrott et al. demonstrated that the incidence of steam pops was 36% when the LI values decreased to >65 Ω from that when stating the RF applications, and the RF applications with steam pops exhibited a significantly greater LI drop than those without (70.3 ± 14 vs. 36.9 ± 24.0 Ω, *p* < 0.0001).^14^ We stopped the RF applications when the LI values dropped below 50 Ω from when they were started, which may be a reason for the absence of audible steam pops. Five RF applications had an overshoot of the LI drop of up to 51–70 Ω after the RF energy deliveries were stopped, but none of the RF applications had any audible steam pops.

### 4.5 Study limitations

This study had several limitations. First, this was a retrospective, non‐randomized, single‐center study that included a limited sample size. A prospective, randomized, multicenter study comparing a CTI‐RFCA guided by CF and LI monitoring and that guided by conventional surrogate markers estimating the lesion formation will be needed to further validate the usefulness of simultaneous CF and LI monitoring during a CTI‐RFCA. Second, this study focused on the acute success of the CF‐ and LI‐guided CTI‐RFCA. The long‐term efficacy of the CTI‐RFCA procedure remains unknown. That is because 11 subjects had clinically detected CTI‐dependent atrial flutters, while the remaining 39 did not have pre‐diagnosed CTI‐dependent atrial flutters and underwent a CTI‐RFCA as part of an AF catheter ablation. Third, the CF and LI values fluctuated with the respiratory movements during the CTI‐RFCA. Finally, we did not evaluate the relationship between the LI and anatomical features of the CTI, such as the presence of a pouch, because not all subjects underwent either right atreiography or intracardiac echocardiography.

## 5 CONCLUSIONS

Effective ablation sites during the CF‐guided CTI‐RFCA had higher initial LI values and greater LI drops than ineffective ablation sites. An absolute LI drop of 21 Ω and percentage LI drop of 10.8% may be appropriate targets for an effective ablation during a CTI‐RFCA using an ablation catheter capable of simultaneous CF and LI monitoring.

## CONFLICT OF INTEREST

The authors declare no conflict of interest for this article. The protocol for this research project has been approved by a suitably constituted Ethics Committee of Gunma Prefectural Cardiovascular Center (Approval number: 2021015; Approval date: November 12, 2021) and it conforms to the provisions of the Declaration of Helsinki.
